# If Gordon Allport was right, the Likert-type personality scales must be very poor descriptors of personality: he was right

**DOI:** 10.3389/fpsyg.2025.1465742

**Published:** 2025-03-13

**Authors:** Aaro Toomela

**Affiliations:** School of Natural Sciences and Health, Tallinn University, Tallinn, Estonia

**Keywords:** personality, five-factor theory, NEO personality inventory, Allport’s theory of personality, word meaning structure, Likert scale, Likert response format

## Abstract

Gordon Allport suggested that personality has several characteristics: it is inherently inconsistent and contradictory, it is situation-dependent, where situational determinants of personality traits are crucial in structured scenarios, while internal determinants prevail in unstructured situations. Additionally, personality develops not only through maturation, and personality traits cannot be quantitatively expressed as fixed amounts along a continuum of a specific trait. Moreover, “common traits” or general personality dispositions act as useful approximations rather than true personality characteristics. Current theories, particularly the Five-Factor Theory (FFT), challenge all characteristics proposed by Allport. This study provides evidence from two distinct analyses—the reexamination of data from a previous study (*N* = 870) and a replication study (*N* = 1,423)—which evaluate the response patterns of individuals using the widely accepted Revised Neuroticism, Extraversion, Openness Personality Inventory, NEO-PI-R. The findings support all of Allport’s claims. First, it shows that only a subset of NEO-PI-R items are truly transcontextual, while the rest contain situational information. Two indices measuring the consistency of responses, namely the Consistency Index (CI) and the Decisiveness Index (DI), were developed. Second, it was noted that the level of inconsistent and indecisive responses was significantly high. Third, it was found that the consistency and decisiveness of responses were systematically and reliably linked to the presence or absence of situational information in the items and the predominant type of word meaning structure that reflects the level of psychological development. These correlations confirm that inconsistency and indecisiveness do not stem from random or careless responding styles. Consequently, an analysis of the most dubious evidence against the FFT, specifically the NEO-PI-R is the most unlikely test to refute FFT. Furthermore, the results of the current studies suggest that summary scores derived from all questionnaires and inventories utilizing Likert-type response formats may be significantly misleading; the consistency of response patterns must be empirically validated before interpreting summary scores.

## Introduction

Personality testing has evolved into a multi-billion-dollar industry. This type of testing is based on the belief that personality assessments can reveal information about us—along with employers, clinical psychologists, educators, and others. It is typically believed that a limited number of personality types or dimensions (often fewer than 30) help differentiate individuals and provide a supposedly comprehensive overview of personality.

Generally, there are two primary approaches to personality testing. Numerous widely recognized assessments, such as the Myers-Briggs Type Indicator (MBTI) (Briggs Myers et al., 1998; a comprehensive list of abbreviations is provided in Supplementary material), classify individuals into distinct “types.” Conversely, other assessments, such as the NEO-Personality Inventory (NEO-PI) (Costa and McCrae, 1992; Piedmont, 1998), map an individual’s test results along a continuum of personality traits. Furthermore, empirical evidence indicates that various personality testing systems can be combined through statistical data analyses within the framework of the Five-Factor Model, which supports the NEO-PI (McCrae and Costa, 2003, Ch. 3).

Nevertheless, a pertinent question arises: what knowledge can be gained from using such assessments? Undoubtedly, it is possible to predict, to some extent above chance, specific characteristics of individuals, including their potential for job success, their fit within an organization, and the effectiveness of interventions in clinical settings. However, this does not necessarily mean that personality assessments truly reflect the essence of an individual’s character.

### Gordon Allport’s views on personality

Gordon Allport (1897–1967) had a unique understanding of personality that differs significantly from the descriptions offered by several popular personality tests today, such as the NEO-PI. The main concepts of his theory explored in this study are as follows (cf. Allport, 1937, 1961; Allport and Odbert, 1936).

First, Allport suggested that any theory viewing personality as stable, fixed, and invariable is incorrect—personality is always experienced in relation to certain situations. Consequently, *personality must be situation-dependent*.

Second, *personality is inherently inconsistent and contradictory*; individuals can hold opposing attitudes toward the same aspect of the world. In other words, people do not fit neatly into specific types, such as extroverts or introverts. Instead, most individuals are of a “mixed type”; they can be both introverted and extroverted simultaneously under different circumstances. Consequently, people cannot be meaningfully positioned along a continuum of personality dimensions: quantitatively, “moderately extraverted” individuals may exhibit extraversion in many situations, but introversion, albeit less frequently, can be found in other contexts.

Third, the *situational determinants* of personality traits—broad systems of similar action tendencies within individuals—*are more significant in structured situations, while internal determinants dominate in unstructured ones*. Allport stated, “Situational determinants are most crucial where duties and roles, as well as tasks and functions, are clearly defined. Personality determinants gain greater significance when the task is freer, more open, and less structured” (Allport, 1961, p. 179).

In the context of this study, it is essential to consider that the third assumption is linked to the first assumption regarding the situation-dependence of personality. The idea that personality is situation-dependent contrasts with the belief that personality is transcontextual, meaning it is independent of any specific situation. Personality traits can be regarded as entirely individual without reference to any external circumstances. This scenario represents an extreme case of an unstructured situation—one that is devoid of any situational context. However, when a personality trait is associated with even a very general situation, certain constraints on the expression of personality traits come into play. Thus, transcontextually formulated items in personality questionnaires relate to conditions that lack situational structure, whereas items that refer to any external situation, even in the most general terms, inherently include some situational constraints or structure.

Furthermore, *personality evolves over time*. Children tend to be much more “situational,” meaning they modify their behavior according to circumstances, compared to adults. Allport discussed this aspect of personality: “… the fact is that young children are more “situational” than adults. They immerse themselves in the hilarity, fear, or despair of the immediate situation and appear to lack an ‘inner personality” while doing so. We have stated that children are prisoners of their culture; similarly, they are also prisoners of the situation. Adults experience this to a lesser degree” (Allport, 1961, p. 178).

Fifth, personality traits are not fixed or stable; they do not exhibit uniformly in every situation. Instead, traits can be understood as ranges of potential behaviors. In other words, *traits cannot be quantitatively represented as specific amounts along a continuum*.

Finally, the statistical psychologists of his era discussed “unique” or “pure” traits, which were identified through factor analysis. According to these perspectives, it was—and still is today—assumed that all individuals share the same fundamental personality structure. Allport rejected these ideas: personal dispositions do not align with the factors derived from the entire population of subjects—"*common traits” are “at best only convenient approximations”;* they *“pertain only to the abstract average person and not to individual cases.”* [SIC].

### Recent views on personality: five-factor theory

One of the most popular personality theories today is the Five-Factor Theory (FFT) (McCrae and Costa, 1996, 2003, 2008, 2021; McCrae and Sutin, 2018), which contradicts the principles proposed by Allport. It is important to note that FFT is just one of the “Big Five” theories, and other Big Five theories do not necessarily adhere to the same fundamental principles as FFT. It is assumed that personality is generally coherent and consistent; the personality system comprises five basic tendencies or dimensions—Neuroticism, Extraversion, Openness, Agreeableness, and Conscientiousness (also referred to as the “Big Five”)—which are further divided into facets (traits related to a domain) and nuances (subtraits within facets). All three—nuances, facets, and domains—are considered transcontextual. This system is regarded as universal since the NEO-PI results from diverse populations can be statistically aligned into a generally similar five-factor structure. According to FFT, traits are not shaped by life experiences but are influenced solely by biology. Although life experiences may impact personality change, such changes can only be quantitative. The level of a personality dimension can either increase or decrease (Bühler et al., 2024). FFT posits that personality can develop, but this development is viewed as a result of biological maturation or other processes that affect the brain. Additionally, development or change in personality is assumed to be strictly quantitative (Bleidorn et al., 2022; Furnham and Cheng, 2019). Finally, FFT assumes that personality can be measured and that individuals can be meaningfully described in quantitative terms as possessing a certain level of a personality trait.

### Questions asked in this study

Overall, several questions need to be answered regarding our understanding of human nature:

(1) Is personality coherent, or is it inconsistent and contradictory?
(2) Is personality situation-dependent or not?
(3) Do humans behave differently in structured versus unstructured situations?
(4) Is personality shaped solely by maturation, or are there more complex developmental processes at play?
(5) Can personality traits be expressed as specific amounts on a continuum, or is the quantitative assessment misleading?
(6) Finally, are “common traits” reflections of the individuals’ true nature, or are they simply overgeneralized abstractions?

### A theoretical foundation for the novel analytical methods developed for this study

The most stringent way to find answers to these questions is to use a personality test explicitly constructed based on a theory that contradicts Allport’s ideas. Thus, the NEO-PI-Revised was selected to demonstrate that Allport is correct on all counts. This study shows that performance on the NEO-PI-R reflects all the personality characteristics proposed by Allport. Therefore, many underlying theories of personality testing today are questionable. Novel measures were devised to reveal the shortcomings of personality questionnaires.

### Measuring inconsistency of personality

It is acknowledged in the interpretation of the NEO-PI-R results that responses to individual items in the questionnaire do not provide pure measures of the personality domains and facets. Consequently, the items are summed with the assumption that statistical error variance is canceled out, leading to a purer indicator of the underlying trait. It is crucial that a Likert-type response format (often mistakenly referred to as a Likert scale, cf. Likert, 1932) is employed in the inventory. Respondents are asked to select from the options: *strongly disagree, disagree, neutral, agree,* or *strongly agree*. Each response is assigned a numerical value (0–4, respectively), and the responses are summed for each domain separately. In summing the responses, it is assumed that the responses can be regarded as distributed on an ordinal or even interval scale, where each subsequent response level is quantitatively one unit higher than the previous one.

Let us consider two items from the NEO-PI-R (both from the Extraversion dimension): (1) *I do not get much pleasure from chatting with people,* and (2) *I really enjoy talking to people*. Both items reflect the same attitude toward talking to people with opposing wording. In these cases, item (1) is reverse-coded; responses from 0 to 4 are recoded as 4 to 0, respectively. After recoding, a higher sum of the two items indicates a greater degree of extraversion in a person. If someone receives a sum of 4, we would assume they are neutral about talking with others. However, some individuals might respond with “strongly disagree” or “strongly agree” to both items; after reverse coding the first response, this results in a sum of 4 + 0 = 4. Clearly, responding “I really like talking” *and* “I really do not like to chat” contradicts the idea of being neutral about both items. The first pair of answers is internally contradictory, while the second is consistent.

This fact facilitates the measurement of personality inconsistency. Following the reverse coding of negatively worded item responses, all “strongly disagree” and “disagree” responses are assigned a value of “−1”; “neutral/undecided” responses are assigned a value of ‘0”; and “agree” and “strongly agree” responses are assigned a value of “+1.” All items related to the same domain are then aggregated after the recoding, and the absolute value of the sum is divided by the total number of non-neutral responses within that specific domain of the test. The resulting value is known as the *Consistency Index* (CI), which ranges from 0 (indicating absolute inconsistency: an equal number of opposing responses to items designed to measure the same trait) to 1 (indicating absolute consistency: responses indicating *agreement* or disagreement exclusively to items that measure the same trait).

### The middle is not in the middle: how to interpret “neutral” or “undecided” responses?

CI characterizes between-item inconsistency. However, some responses may also reflect within-item inconsistency. These responses relate to “neutral” (“undecided” according to Likert, 1932; “hard to answer” in Estonian tests using the Likert format) answers. When item responses are simply summed, it is assumed that a “neutral” response indicates a middle position between the extremes of the trait being measured by the item. However, “neutral” or “undecided” can carry additional meanings. It may suggest, “It depends, sometimes this, sometimes that,” or it can mean, “I do not have an answer,” “I have no idea,” or “I do not understand the statement,” etc. (Valsiner et al., 2005; Wagoner and Valsiner, 2005). Therefore, the proportion of “undecided” answers may reflect confusion rather than quantitative middle points between extremes or a lack of a clear answer. Consequently, the *Decisiveness Index* (DI), which measures the proportion of “decided” (i.e., agree or disagree) responses from the total number of answers, serves as another indicator of response inconsistency.

Both CI and DI contain potentially valuable insights about test performance. In principle, identical summary scores may obscure highly different levels of consistency and/or decisiveness in responses. The proportion of inconsistent or contradictory responses reveals how much the summary score can be placed on a continuum of a personality dimension: this can only be evaluated when the consistency of responses is high. Otherwise, the test results should be interpreted as evidence of a “mixed-type” personality. In such cases, the summary score is not interpretable. Additionally, elevated levels of DI suggest that the summary score may also be non-interpretable, as a high DI level indicates that many items may carry ambiguous meanings for the respondent.

### Measuring situation dependency

The NEO-PI-R may appear to be an unlikely inventory for studying the effect of situations on responses because its creators designed the items to be situation-independent. Therefore, utilizing the NEO-PI-R to demonstrate that situational dependency is a pervasive characteristic of personality presents a particularly stringent test of the idea. A content analysis of the NEO-PI-R items revealed that they can be categorized into two groups. First, there are items that are truly *transcontextual*; that is, they refer to no specific context. “*I rarely feel fearful or anxious”* and “*I strive to achieve all I can*” belong to this category.

Other items, however, suggest a certain context and specific situation. *Sometimes, I cheat when playing solitaire* (context: playing a game); *I see myself as broad-minded and tolerant of others’ lifestyles* (context: observing people’s lifestyles); and *watching ballet or modern dance bores me* (context: watching ballet) all fit into this *situational* category.

According to Allport’s theoretical ideas, responses to personality questionnaire items should depend on the situation and the level of structure present. Therefore, it can be hypothesized that responses to items containing situational information are likely to be less consistent and less definitive than responses to truly transcontextual items.

### Development involves not only the brain but also social and cultural experiences, highlighting the significance of the dominant type of word meaning structure

Individual differences in information processing are typically associated with the concept of intelligence, which is manifested in the ability to solve various types of problems and to base adaptive behaviors on interactions with the environment. However, another psychological construct, initially proposed by Vygotsky, can be distinguished (though not separated) from the concept of intelligence: the predominant type of word meaning structure (Toomela, 2017, 2020b; Vygotsky, 1934; Vygotsky and Luria, 1994). In his theory of verbal thinking, Vygotsky suggested that there are qualitatively different modalities through which information can be processed in verbal cognition. These forms of thought develop in stages of a hierarchical order, occurring solely through interaction with a culturally structured environment. It is proposed that all individuals will develop what Vygotsky called the everyday conceptual structure of word meaning; however, substantial participation in a specially organized environment, such as formal education, is essential for the development of the so-called scientific (the term “logical” may be more fitting, as this type of thinking can also be applied in non-scientific domains) conceptual structure of verbal thought. Moreover, engagement with logical concepts is not a binary phenomenon; rather, individuals show variability based on their cultural and educational experiences across various domains where they can apply more advanced logical conceptual thought.

According to Vygotsky’s theory, information in everyday conceptual thought is organized based on experiences from the everyday world that are encoded in language. Logical concepts introduce a new way of thinking: the ability to organize thought intralinguistically develops alongside metacognition and the capacity to reason according to the principles of formal logic. It is important to clarify that WMS is not identical to intelligence: WMS relates to *how* verbal thought is structured, while intelligence pertains to the ability to solve problems accurately.

There are many ways to reveal the presence of logical concepts, such as intralinguistic hierarchy or the lack thereof (Luria, 1974, 1979). For instance, word triplets can be used in a task that asks which two words from the triplet “go together” *and* to justify that choice. Individuals who think differently may still select the same pair. For example, in the triplet soup carrot-potato, *they* might conclude that carrot and potato belong together. Differences in the organization of verbal thought underlying their choices are evident in the justifications provided. A logical conceptual thinker might argue that potatoes and carrots go together because *both are vegetables*. Here, the hierarchical term “vegetables” unites various types of vegetables into one category. In contrast, an everyday conceptual thinker may justify this based on practical experience, stating that *potatoes and carrots go together because both grow in a field*.

Metaphorically, DWMS can be seen as a tool for thought; intelligence requires such “tools,” along with special knowledge and skills to apply the “tools” across various thinking and behavior organization areas. More advanced tools, meaning developmentally higher forms of the WMS, enable a more complex organization of thought and either solve problems more efficiently or, in certain domains, address problems that cannot be solved at earlier stages of development.

Theoretically, DWMS should be a fundamental aspect of the human psyche that influences all areas of mental functioning (Toomela, 2010, 2020a,b; Vygotsky, 1934, 1960; Vygotsky and Luria, 1994; Vygotsky and Luria, 1930). Indeed, DWMS has been shown to affect various psychological processes that have been studied. Vygotsky’s group previously found that perception, generalization and abstraction, deduction and inference, reasoning and problem-solving, imagination, and self-analysis, along with self-awareness, are distinctly different and less developed in unschooled everyday conceptual thinkers compared to schooled logical conceptual thinkers (Luria, 1974). More recent studies have shown that certain more visually realistic drawings appear *only* in individuals who primarily think in logical concepts (Toomela, 2002, 2022); the structure of personality, as measured by the NEO-PI-R, is less differentiated in predominantly everyday conceptual thinkers (Toomela, 2003); the complexity of self-descriptions regarding personality is significantly higher in logical conceptual thinkers (Arro, 2010, 2012; Ots, 2010); the quantitative levels of personality dimensions—collectivism, coping styles, aggression, and attitudes toward narcotics—correlate with the results of the DWMS test (Toomela, 2008a); in stressful situations, depression tends to develop especially in everyday conceptual thinkers (Toomela, 2008b). Furthermore, predominantly logical conceptual thinkers tend to perform better on tests of visual-perceptual abilities (Tammik and Toomela, 2013; Toomela et al., 2020b), in identifying crossed-out figures (Murnikov, 2021), on indicators of cognitive reserve (Tammik and Toomela, 2017), in response accuracy during investigative interviews (Kask et al., 2019), and in free recall of previously seen events (Murnikov and Kask, 2021; Suurna et al., 2024), as well as on tests assessing cognitive inhibition and verbal behavior regulation (Toomela et al., 2020a).

In this study, the DWMS test was employed to assess the developmental level of verbal thinking, which is clearly influenced by cultural factors. If Allport was correct in suggesting that personality develops qualitatively rather than solely through brain maturation, then personality structure must be linked to DWMS; everyday conceptual thinkers likely exhibit less consistent personalities. This is because everyday conceptual thought does not facilitate the intralinguistic organization of knowledge, which underpins the emergence of well-defined categories.

## Study 1

In Study 1, we reexamined the results of a previous study (Toomela, 2003) concerning the consistency, decisiveness, and situational dependence of responses on the NEO-PI-R.

### Methods

#### Participants

Participants included 870 native Estonian males from the Estonian military (recruits, noncommissioned officers, officers, and members of the Estonian Defense League) as well as undergraduate students (mean age = 24.64 years, SD = 8.14). Within this group, 255 (29.3%) had primary education (9 years or less), 498 (57.2%) held secondary education (12 years), 62 (7.1%) were first- or second-year university students, and 55 (6.3%) had completed a university degree (Bachelor of Science or Bachelor of Arts in nearly all cases).

#### Measures

##### Personality inventory

Personality was evaluated using the Estonian version of the NEO-PI-R (Costa and McCrae, 1992). The Estonian NEO-PI-R (Kallasmaa et al., 2000) was developed based on the Estonian NEO-PI (Pulver et al., 1995). The NEO-PI-R items assess five primary dimensions of personality: Neuroticism, Extraversion, Openness, Agreeableness, and Conscientiousness. Each of these main dimensions is further divided into six facets, containing eight items per facet. The Estonian version of the NEO-PI-R demonstrates high Cronbach’s alpha scores for both domain and facet scales (0.87–0.93 and 0.56–0.85, respectively). Moreover, the retest reliabilities are satisfactory, as the test–retest correlations over a two-year period ranged from 0.67 to 0.86 for domain scales and 0.48 to 0.81 for facet scales. The Estonian NEO-PI-R also confirms the presence of five factors, which align reasonably well with the original inventory. After Procrustes rotation—adjusting a factor matrix to conform to a target matrix defined by the analyst—an excellent fit with the American target was attained for all five factors.

##### Situational and transcontextual items

Items from the NEO-PI-R were classified into two groups based on the criteria mentioned above. The situational category included items that suggest some external situation or context, while the transcontextual category comprised items that reference no context. All test items were coded by the author and two psychologists who were unaware of the study’s aims. The interrater agreement level (calculated using ReCal, Freelon, 2010) was high: Fleiss’ *κ* (Fleiss, 1971) = 0.77, indicating substantial, nearly perfect agreement (cf. Landis and Koch, 1977). All three judges agreed on 83.3% of the items; for the remaining 16.7%, the author’s code was applied. Of these 40 items, one of the other judges concurred with the author’s code in 38 instances, while both other judges disagreed with the author in only 2 cases. In total, there were 143 items in the situational category and 97 items in the transcontextual category.

##### Consistency index

As mentioned earlier, the CI was established as follows. First, all responses to items phrased negatively regarding their respective dimensions and facets were reverse-coded. “Strongly disagree” and “disagree” responses were given a code of “−1,” “undecided” was coded as “0,” and “agree” and “strongly agree” received a code of “+1.” All items within the same personality dimension of the NEO-PI-R were summed, and the absolute value of this sum was calculated. A higher sum indicates a greater number of responses at one extreme (agree) or the other (disagree) on the Likert response scale. A sum of zero signifies an equal number of responses from both extremes. To calculate the CI for the entire test, all personality dimension sums were combined, and the result was divided by the total number of non-neutral responses for the entire test. This result yields a CI that ranges from 0 (absolute inconsistency: an equal number of opposing responses to items intended to measure the same personality trait) to 1 (absolute consistency: responses that either only agree or only disagree with items measuring the same trait). A higher CI indicates more consistent responses to the test.

The *Situational Consistency Index (SCI)* and the *Transcontextual Consistency Index (TCI)* were calculated separately for situational items and transcontextual items, respectively.

The *Decisiveness Index (DI)* is calculated as the number of decisive responses (all non-neutral responses) divided by the total number of items in the test (240). A higher DI indicates more coherent and consistent responses to the test. The *Situational Decisiveness Index (SDI)* and *Transcontextual Decisiveness Index (TDI)* are calculated separately using situational and transcontextual items, respectively.

##### Dominant type of word meaning structure

An original test, designed based on Luria (1979) suggestions, was created to measure the dominant type of word meaning structure. Three complementary measures of word meaning structure were employed. The first part of the test consisted of *definitions* for eight concepts. Half of these concepts were concrete (*car, hospital*), while the other half were abstract (*republic, revolution*). The second part included nine word *pairs*, where participants were asked to describe the most significant similarity between the concepts. The word pairs varied in the clarity of their similarity; some referred to items in the same category (e.g., dog–cat), while others pertained to objects in complementary relationships (e.g., *head–hat*). The third part consisted of nine *triplets of words*. Participants were tasked with indicating which two words out of three “go together” and explicitly stating why the selected two words are related. All 26 responses were classified into two categories: everyday concepts (coded as 0) or logical concepts (or “hierarchical” concepts, coded as 1). The coding criteria followed those proposed by Luria (1979) (see above for details). Responses were coded by two assistants, and protocols from 50 randomly selected participants were simultaneously assessed by both assistants. Interrater agreement was high, with Cohen’s *κ* = 0.91 when adjusted for chance. Doubtful cases were discussed and coded after consultation between the assistants. A participant’s dominant structure of word meaning was determined by summing all item scores, with a maximum score of 26.

### Results

In this study, we searched for answers to six questions. All the questions address the assumptions underlying modern theories of personality that were explicitly rejected by the founders of personality psychology many decades ago.

#### Is personality inconsistent and contradictory?

To determine this, the CI and DI for the NEO-PI-R were calculated. The CI varied from 0.05 to 1.00 (*N* = 864, with six individuals answering “undecided” to all items, preventing a CI calculation for them), Mean = 0.44 (SD = 0.17), Median = 0.44, and the 90th percentile = 0.67. Only one respondent out of 864 showed complete consistency in their answers. The CI value is clearly related to the ratio of opposing responses to non-neutral answers. A CI of 0 indicates that 50% of responses were either “strongly agree” or “agree,” while the remaining 50% were “strongly disagree” or “disagree.” This means an individual reported being equally introverted and extraverted or neurotic and emotionally stable, for instance. The median CI value (0.44) indicates that half of the group studied (whose results fell below the median) had a response distribution of 72% or less versus 28% or more of opposing responses. Additionally, 90% of the individuals studied had a CI of 0.68 or lower, indicating their response distribution was 84% or less versus 16% or more of opposing responses. We can conclude that a significant number of individuals report conflicting values in personality traits simultaneously, and the number of individuals showing full consistency is nearly nonexistent.

The DI ranged from 0.00 to 1.00; Mean = 0.72 (SD = 0.17), Median = 0.73, 90th percentile = 0.91. Four respondents did not provide any “undecided” responses (DI = 1), while six offered only “undecided” responses (DI = 0). Overall, “undecided” responses were quite common. Fifty percent of participants answered “undecided” to ≥27% of items, and 90% of participants responded “undecided” to ≥9% of items.

CI and DI demonstrated a correlation (*r* = 0.175, *p* < 0.0001), although the effect size was low.

#### Is personality situation-dependent? Does it only develop through maturation, or are more complex developmental processes involved? Are humans different in structured versus less structured situations?

The relationships among DWMS, TCI, SCI, TDI, and SDI can be analyzed to address all these questions.

##### Transcontextual and situational consistency indexes

First, participants were divided into five groups based on the number of hierarchical answers they provided on the DWMS test. The first group consisted of participants who scored more than one standard deviation below the sample mean. The second group included those who scored more than one standard deviation above the mean. The remaining participants, who scored within ± one standard deviation, were divided into three groups of approximately equal size. These groups are labeled H1 (1–6 hierarchical answers), H2 (7–9 hierarchical answers), H3 (10–12 hierarchical answers), H4 (13–15 hierarchical answers), and H5 (16–25 hierarchical answers).

SCI and TCI were then calculated. If personality is situation-dependent and individuals vary in more or less structured situations, then SCI should be significantly lower than TCI. Moreover, if personality development is influenced not only by maturation but also by cultural factors, then the consistency of responses should increase, along with the number of hierarchical and logical conceptual answers on the DWMS test.

**Figure 1:**
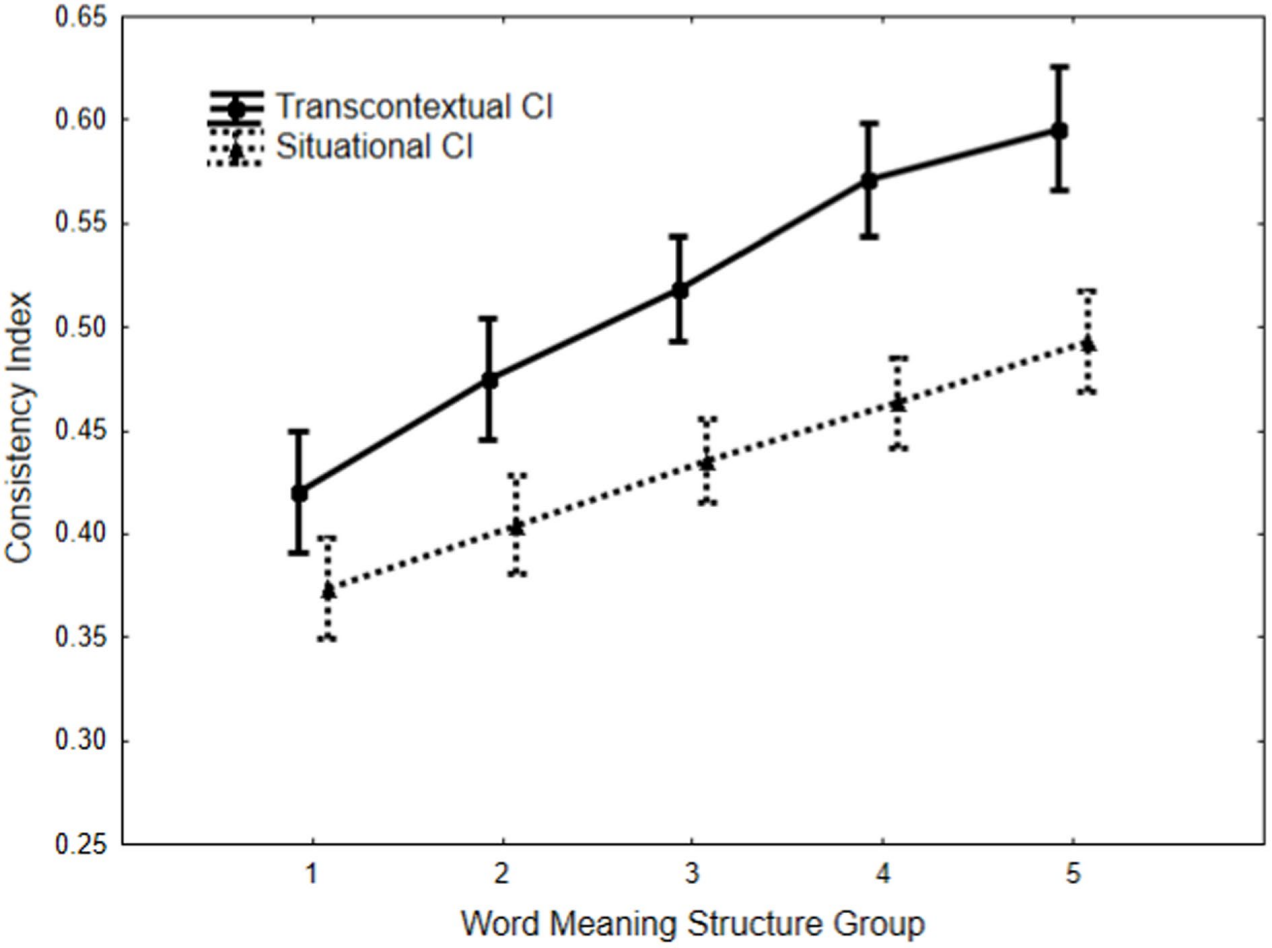
Study 1. Mean levels of transcontextual and situational confidence indices across different groups of word meaning structures. (Vertical bars indicate 0.95 confidence intervals).

The mean levels of the Situational and Transcontextual CI-s for participants in the five-word meaning structure groups are shown in Figure 1. An analysis of variance (ANOVA) revealed significant main effects related to the group, *F*(4, 859) = 22.62, *p* < 0.0001, partial η^2^ = 0.096, CI, *F*(1, 859) = 292.66, *p* < 0.0001, partial η^2^ = 0.254, along with a significant Group × CI interaction, *F*(4, 859) = 5.13, *p* < 0.0005, partial η^2^ = 0.023.

An inspection of Figure 1 reveals that the mean differences between the word meaning structure groups show a systematic pattern: as the level of hierarchical answers increases, both SCI and TCI levels also rise. The overall *post hoc* analysis (Fisher LSD test) revealed that the mean TCI level systematically increased across the DWMS groups: H1 < H2 < H3 < H4 = H5, with all differences between the groups being statistically significant except for H4 and H5 (*p* < 0.015). Additionally, the mean SCI level systematically increased: H1 < H2 < H3 < H4 < H5, where differences between the groups, except for all neighboring pairs (H1 = H2, H2 = H3, H3 = H4, H4 = H5), were statistically significant (*p* < 0.002). The SCI-TCI comparison indicated that SCI was significantly lower than TCI in all DWMS groups (*p* < 0.0001). Furthermore, a significant Group × CI interaction demonstrates that TCI increases more than SCI as the number of hierarchical answers rises.

##### Transcontextual and situational decisiveness indexes

Another approach to addressing the questions of this study is to examine the relationships between DWMS, SDI, and TDI. If personality is indeed situation-dependent and individuals vary in both structured and unstructured contexts, then SDI should be significantly lower than TDI. Additionally, if personality development is not solely based on maturation but is also influenced by cultural factors, then the decisiveness of responses should increase, along with a rise in the number of intralinguistic hierarchical answers on the DWMS test.

**Figure 2:**
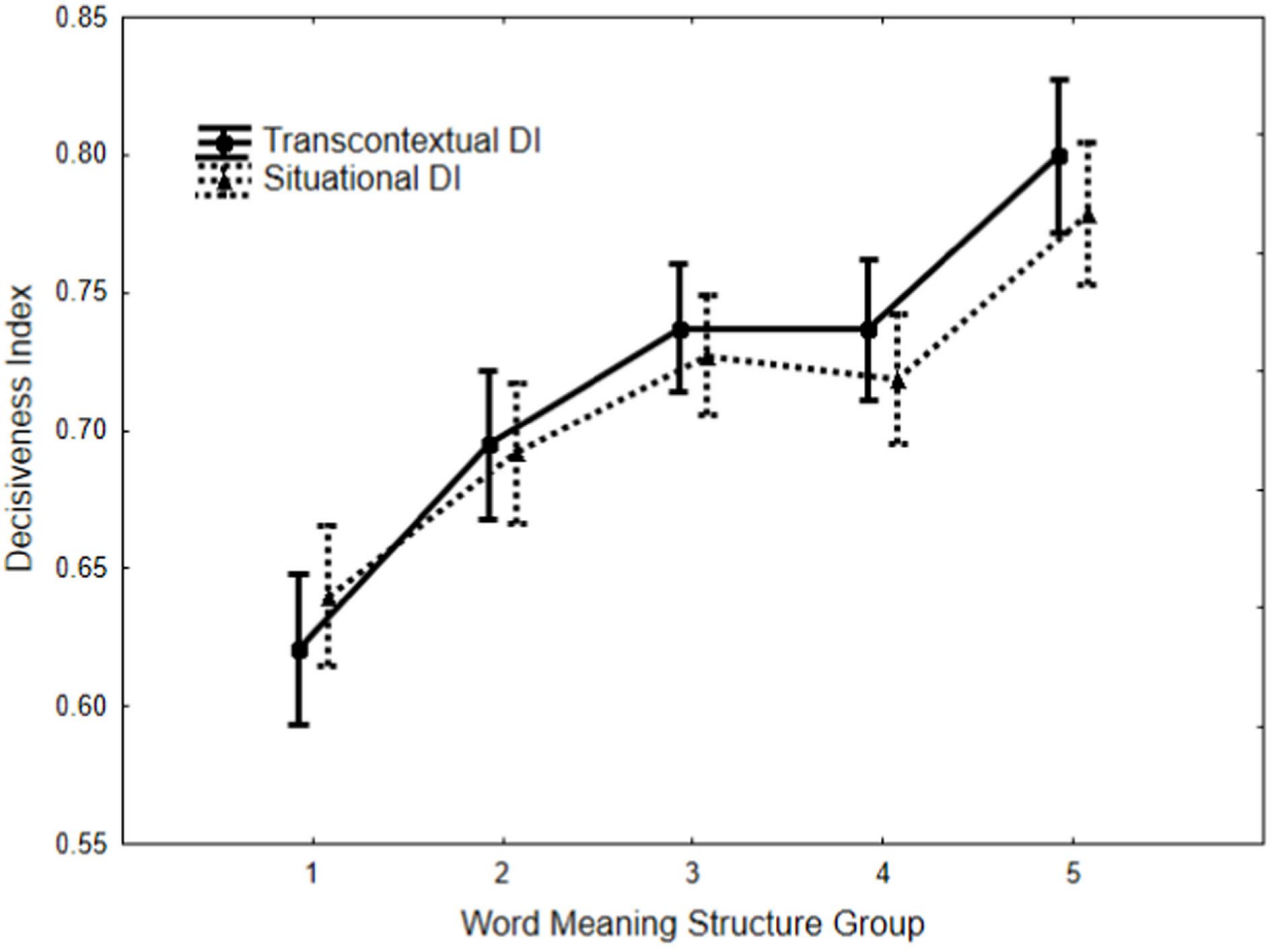
Study 1. The mean levels of transcontextual and situational Decisiveness Indexes among various word meaning structure groups. (Vertical bars denote 0.95 confidence intervals).

The mean levels of the Situational and Transcontextual Discomfort Index (DI) for participants across the five-word meaning structure groups are presented in Figure 2. An analysis of variance (ANOVA) demonstrated significant main effects attributable to the group, *F*(4, 865) = 19.71, *p* < 0.0001, partial η^2^ = 0.084, and DI, *F*(1, 865) = 7.04, *p* < 0.0082, partial η^2^ = 0.008. Furthermore, a significant Group × CI interaction was observed, *F*(4, 865) = 8.16, *p* < 0.0001, partial η^2^ = 0.036.

An examination of Figure 2 shows that the mean differences among the word meaning structure groups follow a systematic pattern: as the level of hierarchical responses increases, so do the levels of both TDI and SDI. Comprehensive *post hoc* analysis using the Fisher LSD test has indicated that the mean level of TDI increases progressively in the following order: H1 < H2 < H3 = H4 < H5. All inter-group differences, except those between H3 and H4, were statistically significant (*p* < 0.02). Similarly, the mean level of SDI also revealed a systematic increase as follows: H1 < H2 < H3 = H4 < H5, with statistically significant differences observed among the groups, except for the pairs (H2 = H4, H3 = H4) at a significance level of *p* < 0.05.

Additionally, TDI was significantly higher than SDI in H3, H4, and H5 (*p* < 0.04); in H1 TDI, it was significantly lower than SDI (*p* < 0.001). The overall DI effect size was small. However, this result is misleading due to the interaction between the group and DI. When H1 was excluded from the analysis, the DI effect size increased considerably (partial η^2^ = 0.031).

##### NEO-PI-R factor structure and response consistency

A series of exploratory maximum likelihood factor analyses (using varimax normalized rotation) were conducted at the facet level of the NEO-PI-R to determine whether the factor structure varies with the CI. Theoretically, as Allport argued, it can be expected that a “universal” Big Five structure emerges only among individuals with a high level of consistency in their responses. A chaotic factor structure should appear if the CI is low because, as demonstrated previously, the CI is situation-dependent. It can be conjectured that individuals with low levels of CI interpret test items more in terms of specific situations personally associated with the items rather than in terms of abstract, transcontextual characteristics of the psyche.

In the original study (Toomela, 2003), which is reanalyzed here, it was found that the factor structure of the NEO-PI-R was systematically related to the DWMS. The expected five-factor structure was not found among groups of individuals who scored low on the DWMS, whereas the expected factor structure emerged among individuals with a high level of hierarchical responses. Proponents of the Five-Factor Theory suggested that the unexpected factor structure in individuals with a low level of hierarchical responses arose because these individuals lacked the cognitive abilities and verbal skills necessary for accurately observing and describing their own personality traits (Allik and McCrae, 2004). The results presented above demonstrate that this interpretation is not supported by the data; the SCI was significantly lower than the TCI even in H1, the group with the lowest level of hierarchical responses. This difference can arise only if the test items are understood and interpreted meaningfully. Nevertheless, to avoid the potential effect of less educated individuals’ tentative inability to accurately observe and describe their own personality traits, the following analyses were conducted only with individuals who have at least a secondary education.

Supplementary Table S1 displays the results of factor analyses using a predetermined number of factors (5) for the entire sample of more educated individuals, as well as for the high-CI (highest tercile) and low-CI (lowest tercile) groups separately. These five factors accounted for 56.57% of the variance in the overall sample. Within the subgroups, five factors accounted for 62.42 and 37.19% of the variance in the CI-high and CI-low groups, respectively. These findings support the hypothesis that only high CI relates to the Big Five personality structure, while low CI corresponds to a less coherent personality framework. An acceptable level of explained variance should be at least 50%, ideally exceeding 60% (Hair et al., 2019; Streiner, 1994). Therefore, the proportion of explained variance is acceptable for the entire sample and even more so for the CI-high group. However, for the CI-low group, as hypothesized, the explained variance was significantly low.

The patterns of factor loadings provided in Supplementary Table S1 indicate that the expected Big Five structure was largely consistent with the original NEO-PI-R across the entire sample. Notably, three facets—N5, N6, and O4—showed higher loadings on a factor different from the one anticipated. Additionally, in the CI-high group, facets N5, N6, and O6 similarly exhibited greater loadings on an alternate factor than expected, while facet A5 loaded approximately equally on two different factors. In contrast, the CI-low group revealed only two distinct dimensions—Neuroticism and Conscientiousness—each with one facet loading incorrectly. The facets related to the other three dimensions (Extraversion, Openness, and Agreeableness) were spread across the remaining three factors, exhibiting no correspondence with the expected structure.

## Study 2

Study 2 was conducted to replicate the findings of Study 1. In Study 2, results from an unpublished study concerning the NEO-PI-R were analyzed. Unlike Study 1, Study 2 employed shorter versions of both the NEO-PI-R and the DWMS test. In other respects, the study design and analytical strategy remained consistent with those of Study 1.

In Study 2, all the main results of Study 1 were replicated. Details of Study 2 are provided in Supplementary material.

### A glimpse at the individual level of analysis

To address the questions of this study, test performance at the individual level should also be analyzed. If personality is not consistent and transcontextual, then there should be high within-individual variability (measured by CI and DI), as well as significant between-subject variability of personality. In particular, individuals with identical scores on a personality inventory should often differ significantly. Four cases from Study 1 will be presented next as examples of this between-subject variability.

### Why x does not equal x

Scores calculated from NEO-PI-R responses are interpreted as accurate quantitative indicators of the underlying constructs. For instance, if two individuals each achieve a score of 66 points on the Neuroticism dimension, the assessment indicates that these individuals possess a similar level of neuroticism. However, if Allport’s assertion holds true—that personality is not as simply quantifiable as assumed by contemporary personality test users—then identical results may not reflect the same underlying traits when examined on an individual basis. This assertion is indeed valid.

The first two examples come from the lower spectrum of the Neuroticism dimension results. A total of 14 individuals achieved a score of 66 on this dimension. This result, placed at the 31st percentile, indicates a moderately low level of neuroticism. However, according to the new measures established in this study, these individuals showed significant differences: the Neuroticism Confidence Interval (calculated only for the Neuroticism dimension) ranged from 0.405 to 0.771, while the Neuroticism Dispersion Index varied from 0.450 to 0.717.

To illustrate aspects of test performance that remain completely hidden in the traditional interpretation of NEO-PI-R results, two cases from these 14 were selected to ensure their DI scores were relatively similar while their CI scores were maximally different. Relevant data about the cases is provided in Table 1. An inspection of Table 1 reveals that Case A displays a relatively consistent response pattern, indicating a moderately low level of neuroticism: out of 35 non-neutral responses, only four items suggest a high level of neuroticism. In contrast, Case B demonstrates that nearly one-third of the non-neutral responses (11 out of 37) indicate that the individual, who is supposedly low in neuroticism, is actually high in it. Consequently, Case B is both neurotic and emotionally stable at the same time, *rather than* just relatively emotionally stable in all aspects. The difference between Cases A and B becomes even clearer when factoring in situational dependence. Case A showed relative consistency with transcontextual neuroticism items, as indicated by the TCI, whereas Case B exhibited inconsistency in both TCI and SCI.

**Table 1:** Descriptive data about cases with identical summary scores on the Neuroticism dimension.

	N^a^	A^b^	Neuroticism					N		A	
			CI	DI	Agree^c^	Neutral	Disagree^c^	TCI	SCI	TCI	SCI
31nd percentile Neuroticism score											
Case A	66	120	0.771	0.583	31	25	4	0.900	0.600	1.000	0.565
Case B	66	113	0.405	0.617	26	23	11	0.429	0.375	0.143	0.323
94th percentile Neuroticism score											
Case C	118	105	0.448	0.483	21	31	8	0.789	0.182	0.200	0.353
Case D	118	121	0.917	0.400	23	36	1	0.833	1.000	0.200	0.536

a N, Neuroticism.

b A, Agreeableness.

c The meaning of “agree” and “disagree” depends on the overall dimension score. For individuals with a low Neuroticism level, the items correspond to low Neuroticism; for those with a high Neuroticism level, the items correspond to high Neuroticism levels.

The same cases are found to exhibit strikingly different response patterns regarding Agreeableness items. Case A demonstrated a relatively high degree of consistency in responses, whereas Case B exhibited a significantly lower level of consistency.

The other two examples arise from the results of the high end of the Neuroticism dimension. Case C and Case D represent two of the six cases that reported high but identical scores on the Neuroticism dimension (94th percentile). Again, as an inspection of Table 1 shows, Case D was relatively consistent in its Neuroticism response pattern, while Case C demonstrated a high level of inconsistency, being, according to the test responses, both highly neurotic and emotionally stable simultaneously. Furthermore, Case D exhibited relatively high consistency with situational Neuroticism items but was almost completely inconsistent according to the Agreeableness TCI measure. In contrast, Case C was nearly completely inconsistent according to the Neuroticism SCI and Agreeableness TCI measures and showed consistency only with the Neuroticism TCI.

Altogether, it can be concluded that identical summary scores on a personality dimension hide substantial differences in the structure of personality.

### Discussion

In this study, responses to six questions were pursued. All the questions examine the nature of personality from different angles and the possibility of characterizing personality using Likert-type scales.

#### Is personality consistent or contradictory?

In contemporary personality theories, it is believed that personality remains stable; for instance, if a person is labeled as neurotic, this trait should appear consistently across various situations and in self-reported personality assessments. However, Allport challenged this idea, asserting that individuals can hold conflicting attitudes about the same aspects of the world.

As indicated by the Consistency Index, which measures between-item consistency, and the Decisiveness Index, which measures within-item consistency, the responses to the NEO-PI-R were notably *in*consistent in this study. From the perspective of the Five-Factor Theory, this inconsistency in responding can be attributed to the low intellectual abilities of some respondents (Allik and McCrae, 2004). Additionally, some studies interpret response inconsistency as careless, random, insufficient effort, or indiscriminate. In these studies, it is assumed that inconsistent response patterns reflect a disregard for the actual item content and distort the “true” factor structure, reliability, and validity of psychological measures (e.g., Arias et al., 2020; Marjanovic and Holden, 2019; Schroeders et al., 2021).

However, evidence suggests that inconsistent responses from a particular theoretical perspective do not inherently reflect carelessness, lack of effort, or inadequate cognitive skills. If respondents can justify their choices, their responses would be context-dependent instead of inconsistent. Studies have demonstrated that individuals’ answers to questionnaire items, including the NEO-PI-R, tend to be coherent based on how they interpret the questions, even if response patterns diverge from the underlying theory of the questionnaire (cf. Arro, 2013; Valsiner et al., 2005; Wagoner and Valsiner, 2005). Our results, which will be discussed in the following sections, also show that response patterns that do not align with the FFT are not necessarily inconsistent with the respondents’ perspective.

Furthermore, we found that the factor structure of the responses aligned relatively well with the Big Five model only in a subsample of individuals with high cognitive intelligence (CI). In contrast, for individuals with low CI, the factor structure of the responses did not correspond to the theoretically expected model. Previous research has shown that the factor structure of NEO-PI-R responses is not consistent across different groups. For instance, it varies alongside the dominant type of the WMS (Toomela, 2003). The unexpected findings from the Five-Factor Theory perspective in that study were attributed to a lack of cognitive abilities and verbal skills among individuals with a primarily everyday conceptual thinking style (Allik and McCrae, 2004). However, this explanation does not pertain to the analyses conducted in this study, as individuals with low levels of education (less than secondary education, i.e., fewer than 11 years) were excluded from these analyses.

Before delving deeper, it is crucial to address some potential issues in interpreting the results of factor analyses. One possible explanation for the differences seen in exploratory factor analyses of the NEO-PI-R results is that the NEO-PI-R may not exhibit a simple structure. Consequently, the placement of the factors under varimax rotation can appear somewhat arbitrary. Moreover, confirmatory factor analysis might also produce questionable results; thus, confirmatory analyses employing orthogonal Procrustes rotation should be applied (McCrae et al., 1996). A reanalysis of Toomela (2003) findings, using targeted rotation, indicated that the expected American normative target structure could generally be replicated, except for the predominantly everyday conceptual thinkers, who were characterized by the proponents of the FFT (Allik and McCrae, 2004) as possessing low cognitive abilities and verbal skills. Nonetheless, the validity of using targeted rotation is highly controversial. The authors of the NEO-PI-R maintain that the American normative target structure should be used, and any exploratory factor analysis results that do not conform to this model should be adjusted toward the American target. However, there is no justification for assuming that the American normative target is the “correct” one. Whether Matrix A is rotated toward Matrix B or vice versa, there is only one closest positioning of the two sets of variables (McCrae et al., 1996, p. 559). Thus, Matrix B could equally act as a “correct” target toward which the American normative structure is rotated.

In current studies, approximately half of the facets show the highest loading on unexpected factors among subsamples of individuals with low cognitive intelligence (CI). Altho.ugh Procrustes rotation indicates a sufficiently high level of congruence among the sets of variables, it remains unclear which matrix accurately reflects the “true” structure of the variables. Indeed, the frequent use of Procrustes rotation across various adaptations of the NEO-PI-R globally suggests that a universal structure may not exist.

Overall, the low levels of consistency and decisiveness in responses observed in this study, along with the finding that the test’s factor structure varies significantly depending on the CI level, demonstrate that the summary scores of the test cannot be meaningfully interpreted. Superficially identical scores obscure individual differences in CI and/or DI levels, suggesting that the interpretations of these identical totals differ on an individual basis. Moreover, it is unclear which items should be aggregated if the factor structures tend to vary significantly among different subgroups of individuals based on CI.

#### Is personality situation-dependent or not?

Allport suggested that personality is situation-dependent. There are no scenarios in which only one dimension of personality is universally manifested; situations are interpreted differently, and various aspects are salient for different individuals (e.g., Arro, 2013; Funder, 2016). Consequently, the variability in the consistency of responses between individuals should be greater when a situation is implied in a question about a particular personality characteristic. Conversely, if no situation is implied—that is, if the inventory item is truly “trans-contextual”—the responses should be more consistent.

Content analysis of the NEO-PI-R items revealed that there are indeed transcontextual items with no implication of any context or external situation whatsoever, and there are items that imply a general context or situation. It is noteworthy that the situations implied in situational items are very general; they simply hint at a type of context. If this general hint regarding some situations is systematically related to the consistency of responses, it can be conjectured that the role of situational information is crucial. Indeed, the response pattern that aligns with Allport’s theory of personality was identified in Study 1 and replicated in Study 2: CI was significantly lower for situational items compared to transcontextual items of the NEO-PI-R. Additionally, transcontextual DI scores were statistically significantly higher than situational DI scores in both studies (although this was only true when the most everyday conceptual DWMS group (H1) was excluded from the analyses).

CI already serves as a measure of response inconsistency based on its calculation method. In contrast, DI can be understood in various ways. The findings from Studies 1 and 2 clearly show that DI measures within-item inconsistency rather than merely indicating the proportion of responses that fall in the middle of the Likert-type scale. It is unfounded to believe that responses in the quantitative midpoint should be chosen more frequently when an item provides situational context.

#### Are humans different in more and less structured situations?

Allport suggested that situational determinants are primarily shaped by situational factors in structured situations, while internal determinants dominate unstructured ones. These concepts align with the current study’s findings: the analysis of CI and DI levels indicated that responses to items suggesting external situations were less consistent with the theoretical Big Five model compared to those that did not imply a situation. Therefore, humans exhibit differences in both structured and unstructured contexts; in the latter, their self-reports align more closely with the Big Five model’s theory. Since *all* human behavior occurs within specific situations, it can be inferred that the five-factor structure is not universal to humanity. Instead, it appears to be an artifact that arises when situational cues are minimized or eliminated from questionnaires.

#### Does personality develop only through maturation, or are there more complex developmental processes involved?

Gordon Allport posited that personality is not entirely shaped by an individual’s biological composition; he stated, “Any theory that regards personality as stable, fixed, invariable, is wrong” (Allport, 1961, p. 175). Moreover, while many factors influencing personality are innate, personality itself is not inherited; a newborn infant lacks personality. Personality emerges and develops when “the original stream of activity meets the environment, acting upon it and being acted upon by it, do the first habits, conscious desires, and incipient traits emerge” (Allport, 1937, p. 122).

Psychic development can be described in various ways. According to FFT, personality traits develop as a result of intrinsic maturation (McCrae and Costa, 2008). Following this concept, personality change is often examined in relation to age. However, age is a nonspecific variable that fails to capture the essence of psychological changes or the processes underlying those changes. True measures of development can only be the properties of the psyche that may or may not emerge over time. The human psyche is a phenomenon that arises from the interaction between the individual and their environment (Toomela, 2020a). This suggests that certain developmental changes in the psyche are only possible when the individual’s environment is structured in a specific way. One cannot learn traditions without a social environment, nor can one learn a language without interacting with individuals who use that language. There is no reason to assume that personality changes occur solely due to biological maturation; personality change is also influenced by environmental factors, including cultural context (Bleidorn et al., 2018; Bleidorn et al., 2009).

Another question is what psychic processes underlie personality changes. One possibility is fundamentally behavioristic: environmental factors “make” personality change. This is how environmental “influences” are typically conceptualized in studies of personality change. However, there is strong empirical evidence (see introduction) suggesting that humans learn not only knowledge and skills but also qualitatively different ways of thinking and organizing personal experiences (see the theory, Toomela, 2017; see also Luria, 1974, 1979; Vygotsky, 1934; Vygotsky and Luria, 1994). The various ways of organizing experiences of the world, as reflected in the dominant type of word meaning structure, underlie all aspects of the psyche—theoretically, DWMS must also influence personality development (see empirical support, Arro, 2010, 2012; Toomela, 2003, 2008a).

In the present study, we examined the potential role of the DWMS in the development of consistency in self-descriptions of personality. Theoretically, individuals who primarily think in everyday concepts should display a less coherent and more contradictory personality structure. This hypothesis was supported: both CI and DI increased significantly with the number of logical conceptual responses on the DWMS test. Therefore, we can conclude that as logical conceptual thinking progresses, personality structure increasingly resembles the statistical Big Five model, even at the item level of analysis.

One important conclusion from these results is that personality development cannot be understood as a change in the level of responses to items, facets, or domains. Instead, it involves changes within domains and facets regarding the relative positions of answers to different items. Thus, the entire structure of personality evolves during development.

#### Can personality traits be represented as specific amounts on a continuum, or is quantitative assessment misleading?

A negative response to this question partly stems from the results discussed thus far. First, substantial intra-individual inconsistency exists in the responses to the NEO-PI-R, as indicated by both CI and DI. This inconsistency also varies significantly between individuals. Therefore, there are considerable intra-individual and between-individual differences in the meanings of the summary scores of the facet and domain scales. Examples of these differences are outlined in Table 1. Those who believe that the summary scores accurately measure the number of personality dimensions or facets overlook the possibility of inconsistent responses, which is extremely common.

Second, we discovered that responses to the inventory vary significantly depending on whether an item includes contextual hints or is truly transcontextual. Consequently, responses to situational and transcontextual items also differ in quality. This poses another challenge in interpreting the quantitative summary scores: results from different inventories or the short and long versions of the same inventory may not be comparable if the proportions of situational items vary. Moreover, even when the same inventory is used, there is—depending on the levels of DWMS and CI—variability among individuals in their responses to situational and transcontextual items. As a result, the proportion of situational items in a questionnaire affects individuals differently, leading to varied interpretations of the summary scores.

Third, the factor structure of responses on the NEO-PI-R varied significantly based on the level of CI: the anticipated Big Five structure appeared in individuals with relatively high response consistency but was absent in those with low CI. In the latter group, the factor structure not only became non-interpretable, but the level of explained variance also dropped considerably. Consequently, sums of facet scores intended to represent the same general domain of personality may convey different meanings across various groups of individuals. Importantly, the unexpected factor structure from the FFT perspective in the CI-low group cannot be attributed to education level, as analyses were conducted with individuals who possessed at least a secondary education. Moreover, the results of factor analyses cannot be attributed to the inventories used, as the expected factor structures were revealed in some subgroups.

Overall, it can be concluded that personality traits are not distributed along a quantitative continuum; characterizing personality as a collection of certain amounts of domains or facets does not reflect the true nature of personality.

#### Are “common traits” expressions of individuals’ true nature, or are they rather overgeneralized abstractions?

In the framework of FFT, it is proposed that the structural representation of the Big Five personality traits found in personality inventories arises from the existence of inherent Basic Tendencies—hypothetical psychological features that characterize an individual’s personality. These Basic Tendencies, which cannot be measured directly, are typically revealed through the use of personality questionnaires. While the individual items within these questionnaires may not serve as perfect indicators of the Basic Tendencies, the cumulative results of numerous items are intended to more accurately reflect personality dimensions, as the aggregated score is believed to comprise a higher degree of true score variance. Additionally, the unique meanings of the Characteristic Adaptations linked to each item are expected to offset one another, thereby producing a more refined indicator of the underlying trait. The identification of items for summation is conducted through factor analysis.

Allport suggested that the “common traits” identified through factor analysis, referred to as Basic Tendencies in the language of the FFT, are abstractions that do not describe individuals; they only apply to an abstract average person. The results of the current studies further support Allport’s position. First, the anticipated Big Five structure of the response patterns emerged reasonably well only in the entire sample (specifically, Study 1) and within the sample of individuals with a high level of CI (Studies 1 and 2). In both studies, the Big Five structure was absent in samples of educated individuals with low CI.

This is not the first study to demonstrate that the Big Five structure does not characterize significant subsamples of human populations. In FFT, it is asserted that the Big Five personality traits are culturally universal (McCrae and Costa, 1997; McCrae et al., 2005). However, the studies that allegedly support the cultural universality of the Big Five are fundamentally flawed. The Five-Factor Model has been examined in numerous countries and languages; however, the samples in these studies have primarily consisted of young, relatively highly educated participants. One method to validate the cultural universality of the Big Five would be to examine societies that are not WEIRD, which stands for Western, Educated, Industrialized, Rich, and Democratic. It is already well established that WEIRD societies, or subsamples of populations from different countries, do not represent the human species in all its diversity (Henrich et al., 2010).

In a study of primarily illiterate indigenous Tsimane society in Bolivia, the personality structure of the respondents did not resemble the Big Five at all, even after Procrustes rotation analysis (Gurven et al., 2013; van der Linden et al., 2018). Furthermore, there is increasing evidence that personality structures differentiate with the increasing complexity of society, known as “niche diversity.” The Big Five personality structure appears to emerge only in more complex WEIRD societies (Durkee et al., 2022; Smaldino et al., 2019).

In fact, the data from one of the most significant studies that supposedly supports the cultural universality of the Big Five (McCrae et al., 2005) aligns with the concept that at least one aspect of WEIRD—specifically, education—differs along with the differentiation of the Big Five across different cultures. In that study, the educational levels of the sampled populations were exceptionally high in all 47 of the participating countries (out of 50, with education levels not provided for three countries). Despite limited variability in educational levels, there were notable correlations with medium effect sizes (correlations of 0.30 or higher according to Cohen, 1988, p. 80) between the levels of education (provided by McCrae et al., 2005, Table 1) and the congruence coefficients found in the factor analyses with Procrustes rotation (provided by McCrae et al., 2005, Table 2) were statistically significant. The correlations between the average levels of education and the congruence coefficients were statistically significant for N (*r* = 0.29, *p* = 0.048), E (*r* = 0.30, *p* = 0.039), and O (*r* = 0.30, *p* = 0.040). Additionally, two correlations did not reach the commonly accepted level of significance: A (*r* = 0.27, *p* = 0.064) and the total congruence coefficient (*r* = 0.27, *p* = 0.071). Only the correlation for C was insignificant (*r* = 0.08, *p* = 0.59).

Cultures are not internally homogeneous; instead, the levels of education, and consequently the Developmental Worldview Model Structures (DWMS), show significant variability across different cultures. Studies claiming to demonstrate the cultural universality of the Big Five personality traits have mostly included individuals with relatively high levels of education. However, if a broad range of educational variability is represented in the study, it may be concluded that the Big Five structure only characterizes those subgroups that are educated and possess more advanced levels of the DWMS. In contrast, among subgroups with lower educational attainment, which mainly consist of everyday conceptual thinkers, the expected Big Five structure, as suggested by the Five Factor Theory (FFT), does not appear (Toomela, 2003).

The DWMS framework qualitatively identifies distinct methodologies through which individuals systematically organize their experiences, including their cognitive processes. It is clear that this characteristic alone cannot fully explain the complexities of personality organization; the content of these experiences is also important. Research has shown that, even among educated university students, the universality of the Big Five personality structure is not definitive. The alignment with the Big Five model is notably stronger among European American and highly acculturated Asian American students than among their less acculturated peers. Additionally, within the same cohort, the factorial structure of personality test results demonstrated variation in relation to the concept of loss of face (Eap et al., 2008).

These findings also indicate that the cultural universality of personality traits among educated individuals does not prove these traits to be human universals. There is growing evidence suggesting that educated individuals from different countries may resemble one another more than those with lower levels of formal education in their respective countries (e.g., Toomela, 2020a,b). The formal education system not only fosters the development of rational facts and skills but also nurtures values, attitudes, identity, and other aspects of the human mind. Formal education systems are becoming increasingly similar across the globe. This trend explains how it is possible to compare educational systems from various countries using measurement instruments such as PISA (Programme for International Student Assessment; more than 80 countries participated in 2022) or TIMSS (Trends in International Mathematics and Science Study; more than 60 countries participated in 2019). Thus, the globalization of the Western-style formal education system contributes to a reduction in cultural differences worldwide.

Overall, substantial evidence exists, even from studies that allegedly support the cultural universality of the Big Five, indicating that the NEOAC five-factor structure is *not* a human universal. The earlier studies mentioned above demonstrate the non-universality of the Big Five at the structural level of analysis. The results of the current study reinforce this conclusion at the item level of analysis: the consistency and decisiveness of responses, measured by CI and DI, respectively, are notably low, particularly among individuals who predominantly rely on less developed forms of verbal thinking.

#### Likert scale and Likert response format

The results of the two studies detailed in this research suggest that there is no basis for assuming that a questionnaire using a Likert-type response format can automatically lead to the construction of a meaningful Likert scale from the items. If the responses are inconsistent from a theoretical standpoint, as demonstrated in the two studies presented here, then summary scores may be misleading and psychologically unjustified. There is no reason to believe that inconsistent responses occur only with the personality questionnaire examined in this study. Therefore, it is prudent to exercise caution regarding the results of all research utilizing Likert scales.

#### Future directions and limitations of the current studies

The results of the two studies reported here demonstrate that the current methods of measuring personality may be highly questionable. Clearly, proposing a comprehensive research program based on a single empirical article is not feasible. Nevertheless, some ideas can be suggested that highlight the limitations of this current study. First, qualitative studies are needed to better understand what the various aspects of personality tests mean for respondents. The response format is likely interpreted differently by different individuals, but the specifics and whether systematic patterns of interpretation exist remain unknown. Furthermore, the interpretation of the items should be examined. For example, it can be speculated that predominantly everyday conceptual thinkers may “translate” transcontextual items into their own situationally organized worlds. The situations and their meanings can also vary in interpretation. Additionally, the strategies for data analysis may need adjustment. If significant inter-individual variability underlies identical summary scores, then group-level analyses should be paired with person-oriented data analyses. Lastly, the results from the studies reported here suggest that CI-s and DI-s should be established for each individual to gain a better understanding of a test’s summary scores.

#### Some clinical implications

There have been increasing suggestions that the Five-Factor Model (FFM) is a reliable theory worthy of application in clinical psychology and medicine (e.g., Costa and McCrae, 1986; Redelmeier et al., 2021; Widiger and Mccabe, 2020). More details are unnecessary here. FFM would be beneficial if there were assurance that the personality structure aligns with the model and that the fundamental principles of the personality theory underlying FFM are valid. The findings from the studies presented here indicate that the evidence supporting FFM is far from satisfactory. In most cases, there is no five-factor structure corresponding to the FFM. The high levels of inconsistency and indecisiveness in responses that characterized nearly all participants in the studies also suggest that NEO-PI-R results should not be interpreted as they currently are. Additional studies are necessary to determine whether FFM has any practical application in clinical psychology and medicine.

## Conclusion

Analyzing response patterns on the NEO-PI-R leads to the conclusion that Gordon Allport’s ideas about the nature of personality are correct. Personality is inherently inconsistent and contradictory; it is dependent on the situation, with situational determinants of personality traits being more significant in structured contexts, while internal, intralinguistic determinants dominate in unstructured ones. The results of this study show that personality development involves not only maturation but also socio-cultural factors, characterized by the predominant type of word meaning structure in this research, which shapes personality’s structure. Additionally, personality traits cannot be expressed quantitatively as fixed amounts on a continuum of traits. Therefore, “common traits” or general personality dispositions are useful approximations but not true characteristics of personality—at least for the majority of individuals. There may, however, be a “WEIRD” minority whose personalities are organized into general domains. Furthermore, the findings of this study have much broader implications. There is no reason to believe that response patterns are inconsistent solely in the context of the NEO-PI-R. Summary scores of Likert scales on all questionnaires and inventories utilizing Likert-type response formats could be highly misleading; the consistency of response patterns must be empirically validated before interpreting summary scores.

## Data Availability

The raw data supporting the conclusions of this article will be made available by the authors, without undue reservation.
