# Supplementary material for: If Gordon Allport was right, the Likert-type personality scales must be very poor descriptors of personality: he was right
Source: Front Psychol. 2025 Mar 13;16:1465742. doi: 10.3389/fpsyg.2025.1465742 (PMC11966457; doi:10.3389/fpsyg.2025.1465742)
Supplement: Supplementary file 1 [file Data_Sheet_1.pdf]

Supplementary Material

List of abbreviations

Abbreviation	Definition
CI	Consistency Index
DI	Decisiveness Index
DWMS	Dominant Type of Word Meaning Structure
FFT	Five-Factor Theory
MBTI	Myers-Briggs Type Indicator
NEO-PI-R	Revised Neuroticism, Extraversion, Openness Personality Inventory
SCI	Situational Consistency Index
SDI	Situational Decisiveness Index
TCI	Transcontextual Consistency Index
TDI	Transcontextual Decisiveness Index

Supplementary Table 1

Study 1. Factor loadings of NEO-PI-R scales after varimax normalized rotation, for entire sample and for high-CI and low-CI groups

NEO-PI-R scales[b]	Entire sample[a] (N = 712)					CI-high[c] (N = 262)					CI-low[c] (N = 205)				
	1	2	3	4	5	1	2	3	4	5	1	2	3	4	5
Neuroticism (N)															
N1	-.42	-.19	-.03	**-.76**	.05	-.28	.07	-.05	**-.74**	-.38	-.05	**.72**	.10	.07	-.06
N2	-.44	-.14	-.23	**-.66**	-.04	-.39	-.02	-.30	**-.59**	-.25	-.09	**.65**	.27	-.01	.17
N3	**-.45**	-.30	-.03	**-.71**	.03	-.31	.04	-.06	**-.64**	**-.55**	-.10	**.75**	-.11	.03	-.08
N4	-.42	-.30	.03	**-.62**	-.01	-.32	-.04	.00	**-.57**	**-.45**	-.18	**.56**	-.19	.25	-.02
N5	**-.66**	.09	-.14	-.31	.10	**-.69**	.11	-.13	-.31	.07	-.41	.13	.14	.39	.08
N6	**-.64**	-.35	.01	-.44	-.19	**-.54**	-.17	-.02	**-.46**	**-.54**	-.44	**.45**	-.17	-.38	.20
Extraversion (E)															
E1	.25	**.67**	.28	.18	.18	.27	.19	.28	.16	**.74**	.10	.08	.12	.44	.14
E2	.10	**.70**	.07	.26	-.07	.13	.03	.07	.21	**.75**	.00	-.16	.06	.10	.32
E3	.41	**.59**	-.29	.22	.21	.38	.20	-.20	.19	**.71**	.30	.03	**.65**	.13	.01
E4	.42	**.63**	-.08	.09	.09	.33	.11	-.04	.12	**.72**	.37	.19	.39	-.00	**.59**
E5	.14	**.69**	-.11	.05	.12	.15	.15	-.17	.13	**.73**	.13	.10	.17	.31	.33
E6	.04	**.65**	.11	.37	.24	-.03	.22	.10	.25	**.77**	-.04	-.33	.28	**.46**	.10
Openness to Experience (O)															
O1	-.35	.09	-.00	.04	**.58**	-.29	**.69**	.01	-.01	.13	-.30	.04	.04	.40	-.19
O2	.13	-.00	.09	.03	**.58**	.07	**.73**	.11	-.04	.01	.08	.10	.22	.17	-.20
O3	.07	.27	.14	-.04	**.61**	.09	**.63**	.08	-.16	.31	.12	-.05	.18	**.55**	-.07
O4	-.09	.18	.03	.36	.21	-.19	.34	.00	.26	.22	-.23	-.25	.02	-.04	.20
O5	.19	.07	-.12	.17	**.62**	.15	**.81**	-.11	.19	.07	.03	.02	.16	.04	-.36
O6	-.14	.05	.05	.41	.34	-.26	.37	.05	.40	.09	-.24	-.34	-.04	.20	-.06
Agreeableness (A)															
A1	.18	.19	**.50**	.29	.16	.04	.12	**.60**	.23	.21	.07	-.16	-.15	.23	.02
A2	.24	-.22	**.60**	.04	-.08	.15	-.12	**.67**	-.07	-.27	.08	.06	-.43	-.05	-.00
A3	.16	.28	**.62**	.10	.20	.19	.19	**.65**	.15	.28	.19	.05	-.29	**.53**	.11
A4	.07	-.18	**.59**	.16	-.13	.08	-.16	**.61**	.08	-.17	.00	-.06	**-.58**	-.05	-.06
A5	-.02	-.40	.42	-.14	-.14	-.09	-.14	.44	-.12	-.44	.11	.01	**-.52**	.10	.01
A6	.02	.13	**.56**	-.15	.24	.06	.27	**.61**	-.05	.10	.03	.19	-.33	**.46**	.04
Conscientiousness (C)															
C1	**.72**	.36	.04	.27	.16	**.65**	.14	.04	.24	**.50**	**.62**	-.23	.11	.25	-.17
C2	**.76**	.19	.10	.01	.02	**.79**	-.06	-.00	-.09	.25	**.54**	-.08	.05	.12	.03
C3	**.73**	.13	.31	.06	.06	**.75**	-.01	.24	.11	.17	**.47**	.02	-.26	.40	-.05
C4	**.75**	.35	.04	-.06	.05	**.72**	.03	.04	-.02	**.48**	**.58**	.14	.06	-.00	-.05
C5	**.79**	.28	.11	.17	.05	**.73**	.05	.02	.14	.45	**.70**	-.17	-.21	.06	.14
C6	**.73**	-.09	.15	.19	-.02	**.76**	-.06	.16	.15	.03	.37	-.22	-.05	-.09	**-.57**

Note: Loadings greater than |0.45| (the level considered to be "fair", Comrey & Lee, 1992, p. 243) are printed in bold.

[a] Factors are reported in order of extraction.

[b] Facet names of personality domains: N1: Anxiety; N2: Angry Hostility; N3: Depression; N4: Self-Consciousness; N5: Impulsiveness; N6: Vulnerability; E1: Warmth; E2: Gregariousness;

E3: Assertiveness; E4: Activity; E5: Excitement-Seeking; E6: Positive Emotion; O1: Fantasy; O2: Aesthetics; O3: Feelings; O4: Actions; O5: Ideas; O6: Values; A1: Trust; A2: Straightforwardness; A3: Altruism; A4: Compliance; A5: Modesty; A6: Tender-Mindedness; C1: Competence; C2: Order; C3: Dutifulness; C4: Achievement Striving; C5: Self Discipline; C6: Deliberation

[c] CI-high = highest tercile CI, calculated from the whole sample; CI-low = lowest tercile CI

Study 2

Study 2 was conducted in order to replicate the Study 1. In Study 2, differently from Study 1, short versions of the NEO-PI-R and the DWMS test were used. In other aspects the study design and the strategy of analysis was the same as in Study 1.

Method

Participants

Participants were 1423 native Estonians (1147 males, 276 females, Mean age = 19.85 years, SD = 2.87), among them 919 male members of the Estonian military: recruits, noncommissioned officers and officers; 107 undergraduate students and 397 last year gymnasium students. There were 306 participants with primary education (9 years or less), 846 participants with secondary education (among them 397 last year secondary education students), 263 with secondary and vocational education, and 8 participants with a university degree.

Measures

Personality inventory. Personality was measured with the short Estonian version of the NEO-PI-R. Instead of 240 items there were 90 items (3 items in each of the 30 facets). The items for the short version were selected on the basis of analysis of the full version NEO-PI-R performance. Three items with the highest loadings on each of the facets were selected.

Situational and transcontextual items. The same codes for situational and transcontextual items of the NEO-PI-R were used as in Study 1. Altogether, there were 37 items in the transcontextual category and 53 items in the situational category.

Consistency index (CI), *Transcontextual Consistency index (TCI)*, *Situational Consistency index (SCI)*, *Decisiveness index (DI)*, *Transcontextual Decisiveness index (TDI)* and *Situational Decisiveness index (SDI)* were calculated in the same way as in the Study 1.

Dominant type of the Word Meaning Structure. In Study 2 a short 18-items version of the original 26-item test was used (6 definitions, 6 word pairs, and 6 triplets of words). Answers were coded the same way as in Study 1.

Results

In Study 2 answers to the same six questions were searched for.

Is personality inconsistent and contradictory?

The CI and the DI were calculated for the short version of the NEO-PI-R. The CI varied between 0.10 and 0.93 (N = 1423, no individual responded "undecided" to all items in Study 2), Mean = 0.40 (SD = 0.14), Median = 0.38, 90th percentile = 0.59. There were no respondents who would have been fully consistent in their responses.

Again it can be concluded that a substantial number of individuals report opposite values in personality traits at the same time and the number of individuals who are fully consistent is almost nonexistent.

The DI varied between 0.30 and 1.00; Mean = 0.76 (SD = 0.11), Median = 0.77, 90th percentile = 0.90. Nine respondents did not give any "undecided" responses but no respondent gave only "undecided" responses.

CI and DI were statistically significantly correlated ($r = -0.101$, $p < 0.0001$), but the correlation was negative, not positive as in Study 1; also the effect size was very low.

Is personality situation-dependent or not? Does it develop only through maturation or are there more complex developmental processes involved? Are humans different in more and less structured situations?

Transcontextual and Situational Consistency Indexes. Mean levels of the Situational and Transcontextual CI-s for participants in the five word meaning structure groups are shown in

Supplementary Figure 1. An analysis of variance (ANOVA) revealed significant main effects due to group, F(4, 1418) = 6.41, $p < 0.0001$, partial $\eta^2 = 0.018$, and CI, F(1, 1418) = 412.88, $p < 0.001$, partial $\eta^2 = 0.226$, but no Group × CI interaction, F(4, 1418)=1.24, $p > 0.2$, partial $\eta^2 = 0.003$.

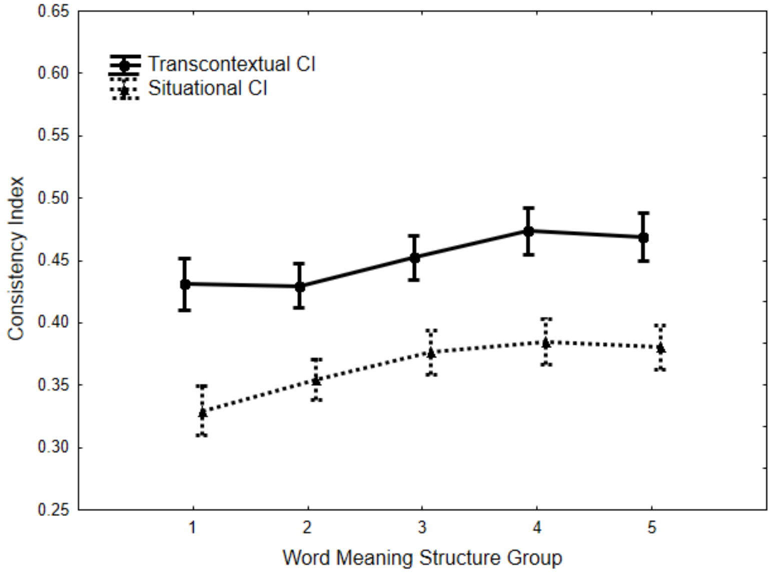

Supplementary Figure 1
Study 2. Mean levels of transcontextual and situational Confidence Indexes in different word meaning structure groups. (Vertical bars denote 0.95 confidence intervals)

Inspection of Supplementary Figure 1 reveals that the mean differences between word meaning structure groups follow a systematic pattern: the higher the level of hierarchical answers, the higher the level of both SCI and TCI even though differences between some neighboring groups were not significant. Overall post hoc analyses (Fisher LSD test) revealed that the two DWMS groups (H1 and H2) were significantly different in TCI from H4 and H5. Thus, overall: H1 = H2 < H3 = H4 = H5 (in case of significant differences, $p < 0.01$ in all cases). In the case of SCI, H1 and H2 were significantly different from H4 and H5 and H1 was also different from H3: H1 = H2 < H3 = H4 = H5 (in case of significant differences $p < 0.02$ in all cases).

SCI-TCI comparison revealed that SCI was significantly lower than TCI in all DWMS groups. Post hoc analyses (Fisher LSD test) confirmed that SCI < TCI in all DWMS groups ($p < 0.0001$ in all cases).

Transcontextual and Situational Decisiveness Indexes. Mean levels of the Situational and Transcontextual DI-s for participants in the five word meaning structure groups are shown in Supplementary Figure 2. An analysis of variance (ANOVA) revealed significant main effects due to group, F(4, 1418) = 9.64, $p < 0.0001$, partial $\eta^2 = 0.026$, and DI, F(1, 1418) = 11.63, $p < 0.0001$, partial $\eta^2 = 0.008$, as well as significant Group × CI interaction, F(4, 1418) = 2.54, $p < 0.039$, partial $\eta^2 = 0.007$.

Inspection of Supplementary Figure 2 reveals that the mean differences between word meaning structure groups follow a systematic pattern which in overall shape is very similar to that found in Study 1. Similarly to Study 1, the higher the level of hierarchical answers, the higher the level of both SDI and TDI. Post hoc analyses (Fisher LSD test) revealed the following

patterns of significant differences between DWMS groups for TDI: H1 = H2 = H3 < H4 = H5 (in case of significant differences, $p < 0.03$ in all cases) and for SCI: H1 = H2 < H3 = H4 = H5 (in case of significant differences, $p < 0.03$ in all cases).

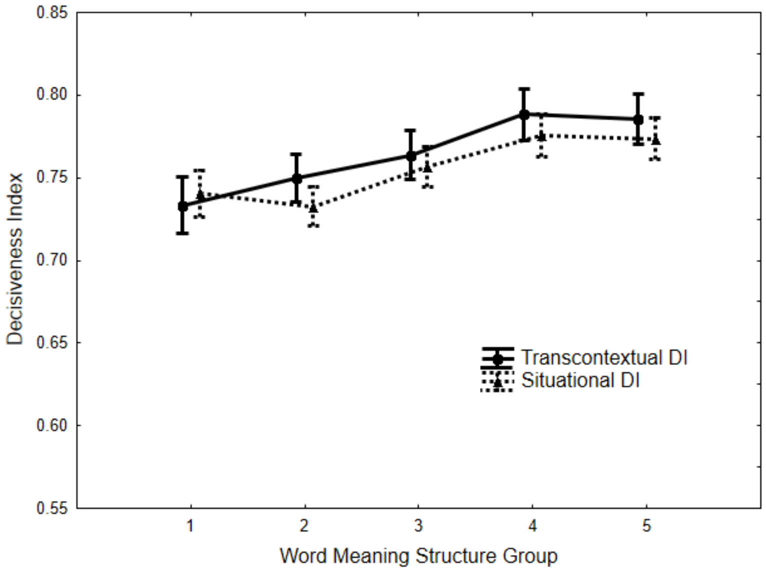

Supplementary Figure 2
Study 2. Mean levels of transcontextual and situational Decisiveness Indexes in different word meaning structure groups. (Vertical bars denote 0.95 confidence intervals)

TDI was also significantly higher than SDI in three DWMS groups (H2, H4 and H5; $p < 0.035$ in all cases; in H1 and H3, $p > 0.17$). Overall DI effect size was very small. This result, however, is misleading because of the group by DI interaction. When H1 was excluded from the analysis, the DI effect size increased considerably (partial $\eta^2 = 0.018$).

NEO-PI-R factor structure and response consistency. In Supplementary Table 2, the results of factor analyses are shown for the entire sample of more educated individuals and for the high CI (highest quartile) and low CI (lowest quartile) groups separately. The five factors accounted for 46.85% of the variance in the entire sample. In the subgroups, five factors accounted for 47.04% and 26.03% of the variance in the CI-high and CI-low groups, respectively. These results are in agreement with the hypothesis that only high CI is associated with the Big Five type personality structure and low CI is associated with a less coherent personality structure. Overall these results of analyses in different groups are similar to the findings in Study 1 with one exception: The levels of explained variances in different groups are lower. Nevertheless, in the entire sample as well as in the CI-high group, the explained variance is close to the acceptable level.

The patterns of factor loadings, provided in Supplementary Table 2, demonstrate that the expected Big Five structure was reasonably homologous to the original NEO-PI-R in the entire sample where still 12 facets (E1, E2, E6, O2, O4, O5, A2, A4, A5, A6, C3, and C5) loaded higher on a different factor than their expected own. In the CI-high group, the factor structure replicated almost perfectly the structure of the original NEO-PI-R; only two facets, O3 and A6, loaded higher on a different factor than their expected own. In the CI-low group, however, majority of factor loadings were low and no clear five-factor structure emerged in the analysis.

Supplementary Table 2

Study 2. Factor loadings of NEO-PI-R scales after varimax normalized rotation, for entire sample and for high-CI and low-CI groups

NEO-PI-R scales[b]	Entire sample[a] (N = 1117)					CI-high[c] (N = 329)					CI-low[c] (N = 271)				
	1	2	3	4	5	1	2	3	4	5	1	2	3	4	5
Neuroticism (N)															
N1	.02	**.82**	.05	-.01	-.08	**-.86**	-.13	-.06	-.10	.00	**.55**	.11	.40	.20	-.31
N2	.05	**.74**	-.12	.02	-.15	**-.65**	-.00	-.06	-.34	.07	.31	.11	**.61**	.05	.16
N3	-.30	**.59**	.22	-.13	.13	**-.77**	-.22	-.08	.08	.00	.32	.10	.01	-.14	.07
N4	.04	**.70**	.18	-.03	-.05	**-.75**	-.19	-.06	.09	-.04	**.50**	-.01	.05	.14	-.21
N5	.17	**.63**	-.13	-.13	.05	**-.55**	.21	-.08	-.08	.09	.10	-.06	.38	-.03	-.02
N6	.14	**.72**	.17	-.21	-.10	**-.70**	-.12	-.33	.10	.05	**.64**	-.02	.19	.19	-.11
Extraversion (E)															
E1	.03	-.24	-.30	.00	**.59**	.10	**.70**	.06	.12	.13	**-.63**	.14	.12	.15	-.11
E2	**.74**	.12	-.27	.00	-.02	.02	**.70**	.07	-.01	.12	**.55**	-.16	.05	-.16	.06
E3	.01	-.00	**-.59**	.18	.01	.21	.40	.23	-.36	-.05	.35	.06	-.10	-.15	-.14
E4	.34	-.12	**-.48**	.17	.27	.10	**.82**	.20	.02	-.05	-.23	.16	.21	.09	.20
E5	.41	-.16	**-.46**	-.04	.16	.17	**.62**	.06	-.23	.19	-.32	-.01	-.04	.05	.03
E6	**.71**	.04	-.29	-.11	-.01	.06	**.76**	-.04	.12	.03	**.48**	-.31	.16	-.08	.04
Openness to Experience (O)															
O1	**.51**	.03	.20	-.15	.05	-.16	-.03	-.06	.12	**.56**	-.08	-.05	-.11	-.18	.28
O2	-.27	.10	-.08	-.07	**.46**	-.05	.04	-.02	.05	**.52**	-.03	.05	.20	**-.60**	-.20
O3	**.64**	.04	.03	-.01	.25	-.10	.42	.03	.36	.39	.04	.02	-.17	.02	.05
O4	-.02	-.04	-.23	-.25	.24	.18	.22	-.24	-.06	.35	.22	.04	-.22	-.12	-.28
O5	.19	.00	.05	.14	.27	-.02	.02	.07	-.02	**.59**	-.21	-.03	.04	.36	.06
O6	**.68**	-.01	.04	.07	-.03	.10	.25	.13	.08	.43	.12	.10	.04	.12	**.46**
Agreeableness (A)															
A1	.32	-.09	.10	.02	**.45**	.07	.20	.06	**.48**	.13	-.08	.12	-.01	-.07	-.23
A2	.12	-.07	**.59**	.05	.05	.04	-.33	-.04	**.56**	.04	-.06	-.10	.30	.10	.18
A3	-.01	-.12	.12	.16	**.65**	.06	.18	.20	**.45**	.17	-.33	.11	-.14	.05	-.22
A4	-.14	-.40	.24	-.06	.20	.14	.02	-.03	**.61**	-.09	.01	.04	-.43	.11	-.07
A5	.33	.11	.43	-.02	.14	-.24	-.10	-.08	.35	.13	-.10	-.00	.14	.05	.19
A6	**.56**	.17	.16	.08	.12	-.08	.08	.12	.35	.38	.08	-.10	.14	-.00	.03
Conscientiousness (C)															
C1	-.15	-.32	-.29	**.61**	.06	.34	.12	**.64**	-.15	-.22	**-.53**	.08	.01	-.10	-.09
C2	.26	.17	-.10	**.60**	.13	-.14	.27	**.62**	.01	.00	.16	**.53**	.02	-.02	.07
C3	**.74**	.06	.07	.40	-.16	.13	.08	**.73**	.10	.18	.05	-.33	.28	.07	.18
C4	.08	-.14	-.20	**.69**	.12	.17	.10	**.72**	-.08	.00	-.26	.38	-.20	.09	-.09
C5	**.72**	.14	.01	.35	-.25	.12	.12	**.80**	.02	.12	**.45**	**-.68**	.19	.20	.10
C6	-.02	-.21	.16	**.64**	.00	.09	-.16	**.57**	.12	-.04	-.23	.08	-.25	-.03	.03

Note: Loadings greater than |0.45| are printed in bold.

[a] Factors are reported in order of extraction.

[b] Facet names of personality domains: See Table 1

[c] CI-high = highest quartile CI, calculated from the whole sample (in Study 1 terciles were used instead due to a smaller size of the sample); CI-low = lowest quartile CI

References

Comrey, A. L., & Lee, H. B. (1992). *A first course in Factor Analysis.* (2nd ed.). New York: Psychology Press.
